# P02-003 - HIDS in a consanguineous family form Saudi Arabia?

**DOI:** 10.1186/1546-0096-11-S1-A110

**Published:** 2013-11-08

**Authors:** Q Zhou, R Sleiman, DL Kastner, I Aksentijevich

**Affiliations:** 1 Inflammatory Disease Section, National Human Genome Research Institute (NHGRI/NIH), Bethesda, MD, USA; 2 Fakeeh Hospital, Jeddah, Saudi Arabia

## Introduction

The daughter of a first-cousin marriage from Saudi Arabia died at age 7y/o of an unexplained periodic fever illness. At two years of age, the proband presented with recurrent fever attacks associated with febrile seizures, severe anemia, septic arthritis, diarrhea, and severe vomiting causing multiple ICU submissions. Considering her ancestry, she was thought to have an FMF-like disease and was subsequently treated with colchicine with partial response.

## Objectives

To identify a disease-causing gene in this family utilizing exome sequencing. Of concern in this family is that the younger siblings have yet to develop the disease.

## Methods

We performed exome sequencing in the unaffected parents, the proband, and two younger siblings. Targeted exon enrichment was performed on 3 μg of DNA extracted from peripheral blood using the SureSelect Human All Exon 50 Mb Kit (~24000 genes, Agilent Technologies).

## Results

We focused our analysis on missense, nonsense, and splice site variants and coding indels, resulting in 11981 variants on average per exome. After excluding common variants (>2%) the analysis yielded a mean of 1415 variants per individual. Finally, 97.5% of these rare variants were eliminated under an autosomal recessive model for the consanguineous family, leaving 38 candidate variants in total. Based on the protein function and the PolyPhen-2 prediction on protein function we selected 14 potentially pathogenic mutations and we confirmed them by Sanger sequencing. Under the assumption that younger siblings are unaffected, the single genotype that stood out was that the proband was homozygous for the V377I *MVK* mutation, while both siblings and the unaffected parents were heterozygous carriers. There were no other mutations in known PF genes identified in this family. The V377I mutation is the most common HIDS-associated mutation and it is considered mild with reduced penetrance. The patient in our study, however, presented with very severe disease but not inconsistent with HIDS. The complexity of her symptoms suggests a role for other modifying alleles.V377I is known as the Dutch-mutation with estimated carrier frequency 1:65 in the Netherlands, however most HIDS patients are compound heterozygous for V377I and another *MVK* mutation. There are no data available on the frequency of V377I in Arab and other Middle Eastern populations. There are very few sporadic reports of HIDS in non-Caucasian populations and typically many patients are followed for years with the diagnosis of familial Mediterranean fever.

## Conclusion

This result should raise awareness for considering HIDS and other uncommon periodic fevers in patients of Middle Eastern ancestry. Alternatively, we may re-analyze the data in the event that one or both of the younger siblings become affected.

## Disclosure of interest

None declared.

